# Combinatorial K-Means Clustering as a Machine Learning Tool Applied to Diabetes Mellitus Type 2

**DOI:** 10.3390/ijerph18041919

**Published:** 2021-02-17

**Authors:** Miroslava Nedyalkova, Sergio Madurga, Vasil Simeonov

**Affiliations:** 1 Department of Chemistry, University of Fribourg, Chemin du Musée 9, 1700 Fribourg, Switzerland; 2 Materials Science and Physical Chemistry Department, Research Institute of Theoretical and Computational Chemistry (IQTCUB), University of Barcelona, C/Martí i Franquès, 08028 Barcelona, Spain; s.madurga@ub.edu; 3 Department of Analytical Chemistry, Faculty of Chemistry and Pharmacy, University of Sofia “St. Kl. Okhridski”, 1, J. Bourchier Blvd., 1164 Sofia, Bulgaria; vsimeonov@chem.uni-sofia.bg

**Keywords:** diabetes mellitus type 2, underlying diseases, multivariate statistics, k-means, classification, descriptors

## Abstract

A new original procedure based on k-means clustering is designed to find the most appropriate clinical variables able to efficiently separate into groups similar patients diagnosed with diabetes mellitus type 2 (DMT2) and underlying diseases (arterial hypertonia (AH), ischemic heart disease (CHD), diabetic polyneuropathy (DPNP), and diabetic microangiopathy (DMA)). Clustering is a machine learning tool for discovering structures in datasets. Clustering has been proven to be efficient for pattern recognition based on clinical records. The considered combinatorial k-means procedure explores all possible k-means clustering with a determined number of descriptors and groups. The predetermined conditions for the partitioning were as follows: every single group of patients included patients with DMT2 and one of the underlying diseases; each subgroup formed in such a way was subject to partitioning into three patterns (good health status, medium health status, and degenerated health status); optimal descriptors for each disease and groups. The selection of the best clustering is obtained through the parameter called global variance, defined as the sum of all variance values of all clinical variables of all the clusters. The best clinical parameters are found by minimizing this global variance. This methodology has to identify a set of variables that are assumed to separate each underlying disease efficiently in three different subgroups of patients. The hierarchical clustering obtained for these four underlying diseases could be used to build groups of patients with correlated clinical data. The proposed methodology gives surmised results from complex data based on a relationship with the health status of the group and draws a picture of the prediction rate of the ongoing health status.

## 1. Introduction

K-means (partitioning clustering) is a method of clustering that is used to classify objects into *k* groups (clusters). This method needs to specify the initial number of the supervised *k* groups.

Each group is normally centered around their mean, and the iterative algorithm is used to minimize the distance between each observation and their corresponding mean. The main objective of the exploratory data analyses for the diabetes data was to reveal the pattern from the data set and build a model based on k-means and hierarchical clustering analyses. The objective was to discover the effects of a group diagnosed with diabetes and assembled by age and evaluating the survival ratio in an efficient manner.

Machine learning (ML) techniques have been used to reveal the predictive patterns for diabetes disease in the last years. The growing density of this problematic topic has provoked researchers to discover robust models based on deep learning (DL) algorithms. However, the application of ML algorithms was applied in solving and mitigating the effect on the society of this problem. The article by Marie-Sainte [1] graphed all the ML and DL techniques based on efforts for diabetes predictions published in the last years. The endorsement is to use these proper classification and prediction models in diabetes disease and improve the robustness of them by developing more complex applicable models.

Similar research has been developed for diabetes estimation created on various algorithms and methods. The work of Anuja et al. [2] offered a diabetes disease classification using a support vector machine (SVM). The methodology was based on k-means clustering in order to find the most appropriate descriptors that are able to separate a predefined number of cases. Rajesh et al. [3] proposed a scheme for diabetes classification based on using C4.5 algorithms. The authors achieved a classification rate of 91% by evaluating the training data through data feature relevance analysis. Aiswarya et al. [4] used decision tree and naive Bayes classifiers for classifying the diabetes diagnosis. Harleen et al. [5] suggested a structure analysis based on a technique for data mining for diabetes disease prediction. The proposed structure includes three main steps which are: pre-processing, feature extraction, and parameter evaluation. In the pre-processing step, the case of lack of data or data with a detected irregularity in the sets were excluded. The patterns that are hidden and the interactions within the data set are tracked in the step for the feature extraction in order to increase the level of the decision making from the obtained results.

Furthermore, the proposed system was evaluated using J48 and naive Bayes, and the achieved rates were 73.8% and 76.3%, respectively. Haq et al. [6] have proposed a study in the frame of machine learning methods for the detection of diabetes. The proposed method has been tested on the diabetes data set, which is a clinical data set designed from the patient’s clinical history. The model validation methods, known as hold out and K-fold, leave one subject out, and performance evaluation metrics including accuracy, specificity, sensitivity, F1-score, receiver operating characteristic curve, and execution time have been used for the validity of the proposed scheme. The decision tree classifier has been used for the classification of the groups of healthy and diabetic subjects. A suitable correlation with experimentally obtained results for the feature selection and a positive effect on the classification performance of the predictive model was observed. The capability of the performed method is due to the fact of the multilevel approach of the selected features set. Krishnaveni et al. [7] proposed six various techniques for the diabetic disease prediction model. The used techniques are discriminant analysis, KNN algorithm, naive Bayes, SVM with linear kernel function, and SVM with RBF kernel function. The results of the study based on the above technique were 76.3% using discriminant analysis, 71.1% using the KNN algorithm, 76.1% using naive Bayes, 74.1% using SVM with linear kernel function, 74.1% using SVM with RBF kernel function. However, several authors [8,9,10] have used various methods in order to obtain the best prediction rate.

In a recent study, Ruy et al. [11] applied a new screening model for estimating undiagnosed diabetes mellitus (UDM) as vital for early medical care. The proposed screening model for estimating UDM among people with a family history of diabetes enabled its validation. The screening model for UDM used data for separated male, female, and combined groups by applying backward stepwise logistic regression analysis. The screening models were established as well as confirmed. In another recent study, Ryu et al. [12] recruited participants with a screening model for undiagnosed diabetes mellitus. Toward undiagnosed DM, the screening model by machine learning methods was applied. The data for 11,456 participants were designated, and the model was evaluated with 4444 participants. The machine learning model displayed a correlation with previous screening models. As explored by the authors, a deep learning model for patients with undiagnosed diabetes could aid in decreasing the effect on the national economic and social effects of the disease.

In this study, we were able to identify an application of a new method that was not only focused on the identification of best descriptors but also the procedure was able to deal effectively with missing data. Extensive research could be found studying the issue of missing data. It has to be noted that the unsuitable treatment of missing data could lead to biased results, as in the case of multiple imputations. Alternatively, we designed an original methodology that does not attempt to fill in the missing data. The calculations with simulated data were performed to address the ability of the method to identify the best descriptors. Additionally, hierarchical cluster analysis was applied to confirm the separation of patients into three clusters. The main purpose of the present study was to check the usefulness of the proposed combinatorial k-means method to accurately classify type-2 diabetic patients diagnosed with underlying diseases with the most appropriate clinical descriptors.

## 2. Materials and Methods

### 2.1. Clinical DMT2 Data with Four Underlying Diseases

Clinical information of 52 objects (diabetes mellitus type 2 (DMT2) patients) having at least one of four underlying diseases (arterial hypertonia (AH), ischemic heart disease (CHD), diabetic polyneuropathy (DPNP), and diabetic microangiopathy (DMA)) was used in this study. From the total of 52 patients, 51 had AH, 48 had CHD, 51 had DPNP, and 47 had DMA. The patients were characterized with some of the following clinical variables: age, height, weight, waist, BMI (body mass index), sysRR, diaRR (systolic and diastolic blood pressure), Hgb (hemoglobin), Er (erythrocytes), Leu (leucocytes), Tro (thrombocytes), creatinine, ALAT, GGT (enzymes alanine aminotransferase, gamma glutamyl transferase), chol (cholesterol), HDL (high-density lipoproteins), TG (triglycerides), HbA1c (hemoglobin A1c), EAG (estimated average glucose), SUE (saturation rate), UA (uric acid), Na, K, Cl, Ca, and P (concentrations of sodium, potassium, chloride, calcium, phosphorus).

Some of the clinical variables are available for all the patients, such as age, but in general, there is some missing information about some values of variables for some of the patients. On average, the patient’s description relies on the information of 23 out of 26 clinical variables having on average two missing values.

### 2.2. Combinatorial K-Means Clustering Analysis Tools

A python tool, named combinatorial k-means clustering analysis tool, was developed to find the separation of cases by k-means clustering using only a small number of descriptors (variables) selected from a large set of descriptors. The combinatorial k-means clustering program was developed under the Python Notebook platform using the following python modules: NumPy, Pandas, Matplotlib, SciPy, and Itertools. The NumPy module was used for efficient numerical calculation of data. The Pandas module was used to read spreadsheet files, having the data of the patients for each underlying disease. Graphical representation of data was performed using the Matplotlib module. The submodule hierarchy of the submodule cluster of SciPy was used to perform k-means clustering. The module Itertools was used to perform all possible combinations of descriptors. The program took all the information from an Excel file chosen by the user. Then, the data were normalized, dividing by the standard deviation of each property. The k-means clustering could be performed by different methods of linkage, as average, centroid, or Ward’s. In this study, the centroid method was selected for all calculations. As a default, the program performed k-means clustering, making all the combinations of three descriptors taken from all properties of the spreadsheet indicated as input data. Optionally, combinations with two descriptors could also be selected. In this study, all possible clustering of three variables into three groups was performed automatically. In order to rank all possible clustering, a property defined as the global variance was computed for each of the k-mean clusterings:

(1) $$varGlobal\;=\;\sum \sum {\mathrm{var}}_{i,k}$$

where var_i,k_ is the variance of the descriptor *i* in the cluster *k*. In this study, Equation (1) is computed having nine terms corresponding to the three descriptors for each of the three clusters.

In order to select a valid clustering, some restrictions were also applied. The random generation of clustering could generate systems having clusters with a non-balanced number of elements. This would generate clusters with one or two elements giving a low variance for their corresponding group. In order to avoid this phenomenon, it is defined that a valid clustering is the case when at least three elements for any of the clusters are involved. The program performed clustering using the combination of three descriptors. For each combination, the appropriate number of cases with available data for the three descriptors under study were selected. In that way, for each clustering, the maximum data available was always used.

**Figure 1:**
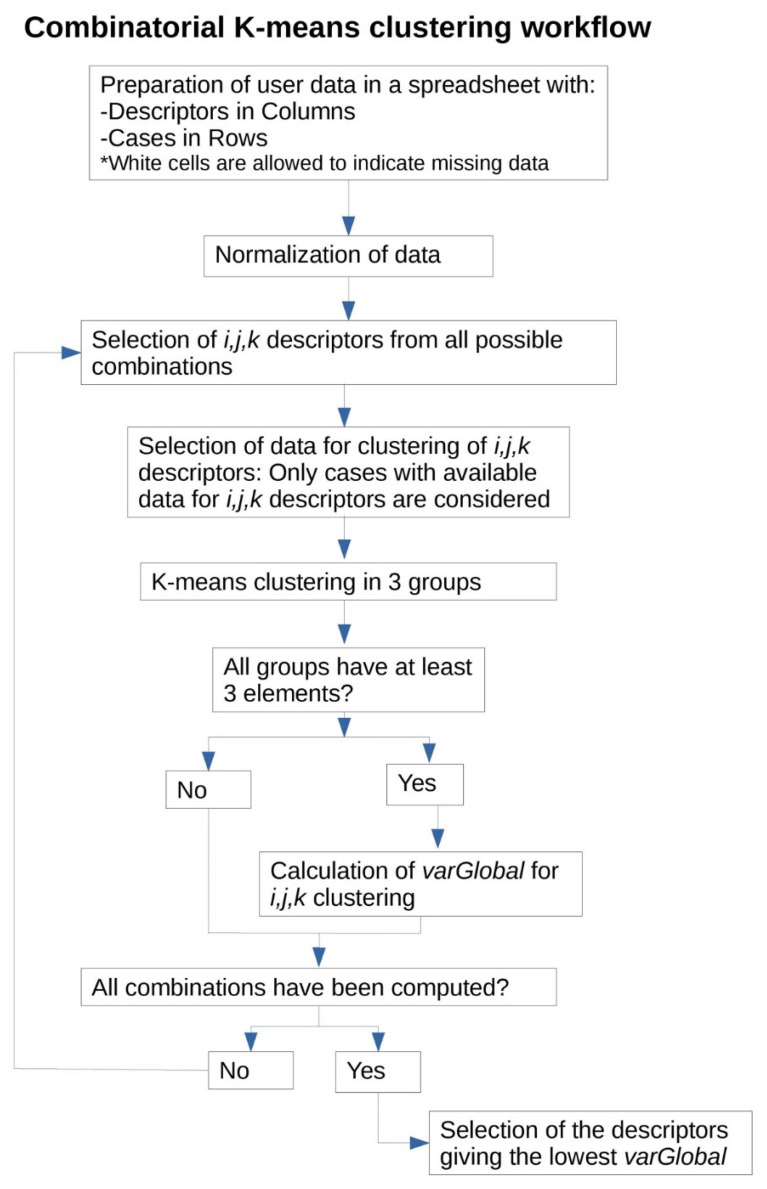
Workflow diagram.

The workflow design diagram of the algorithm is depicted below (Figure 1), and the code is available at https://github.com/smadurga/Combinatorial-K-means-clustering (Accessed on 5 February 2021).

### 2.3. Hierarchical Cluster Analysis (HCA)

The hierarchical clustering analysis (HCA) method is a non-supervised multivariate statistical procedure looking for groups of similar patterns (clusters) within a data set. This method of data exploration consents to the study of the structure of data by the similarities pattern between the objects in the parameter space and between the parameters in the object space. HCA methods vary in terms of the similarity measure applied and the way of clustering. The most commonly applied similarity measure is the Euclidean distance. The applied methods of clustering of objects and variables are well known and widely applied as a valuable algorithm. The common use algorithms are as follows: single linkage method, complete linkage method, average linkage method, and centroid linkage. Ward’s linkage method was applied in achieving the goals in our study. The method requires standardization (normalization) of the raw data (usually by z-standardization), determination of the similarity measures (usually by Euclidean distances), selection of appropriate linkage option and plotting the clusters on a tree-like diagram (dendrogram), and determination of the number of statistically significant clusters by specific test (usually Sneath’s criterion) [13,14]. For clustering, the software product STATISTICA 8.0 was used.

## 3. Results and Discussion

### 3.1. Combinatorial K-Means Clustering with Data Test Values

In order to test the ability of the program to find the most appropriate descriptors for classification, test values having three descriptors that are able to define three clusters and other sets of descriptors having random values were generated.

First, the program generates three clusters with different central values of named *X, Y* and *Z* descriptors. After that, a collection of values corresponding to non-correlated descriptors are also generated. For each run of the combinatorial k-means clustering algorithm, the program should identify the *X, Y* and *Z* descriptors as those defining three clusters. Different runs were performed with different separations of the central values of the three clusters of *X, Y* and Z descriptors. It is expected that when the separation among three groups is large, the *X, Y* and *Z* descriptors are well identified by the combinatorial k-means clustering. However, when the dispersion of the values of the groups is of the order of the separation of central values, these descriptors are probably not identified. To perform this test, a matrix of 30 columns (descriptors) by 45 cases was built. 37 columns were built with random numbers following a normal distribution centered at 1 with a σ of 1. The other three columns (*X*, *Y*, *Z*) were used to define three groups. In particular, 15 cases were randomly generated with a normal distribution centered at point (*p*,0,0) with a dispersion given by σ of 1 for *X*, *Y* and *Z* descriptors. Another 15 cases of *X*, *Y,* and *Z* descriptors were randomly generated with a normal distribution centered at point (0,*p*,0) using σ of 1, and the last 15 cases were centered at (0,0,*p*) using σ of 1. In this set of values, the separation among the three groups in the space of *X*, *Y* and *Z* descriptors is performed using a single value of *p*. It has to be noted that the separation among pairs of groups is only because of the difference in one of the descriptors. The separation of the centroid could be obtained in terms of the parameter *p* being √2 *p*.

The combinatorial k-means clustering study was carried out with different random test set values generated with different values of *p*. For each calculation, three sets of descriptors were selected as the most appropriate for classification using the criterion of the minimum global variance. In each case, it is evaluated if the *X*, *Y* and *Z* descriptors are correctly selected. To obtain a probability of identification of the appropriated descriptors, 500 calculations with different random values were performed. In Table 1, the probability to obtain the *X*, *Y* and *Z* descriptors ranked first from all possible combinations of descriptors (30 × 29 × 28 = 24,360) as a function of the separation of the groups is indicated. It could be seen in Table 1 that when the separation of the groups is small compared to the value of σ, the probability to correctly identify *X*, *Y* and *Z* descriptors is low. At low values of *p*, the randomly generated clusters are so overlapped that they are difficult to be identified from other randomly generated data. When the parameter *p* is three times the value of σ, the 75% of cases are obtained with the *X*, *Y* and *Z* descriptors as the best ones for classification. The success increases to 99% if the separation of the groups is 3.5 times the value of σ. Thus, the separation of the groups with respect to the dispersion of the values in each of the clusters could be an indicator if the selection of the descriptors is appropriate.

### 3.2. Combinatorial K-Means Clustering with Underlying Diseases of DMT2 Patients

**Figure 2:**
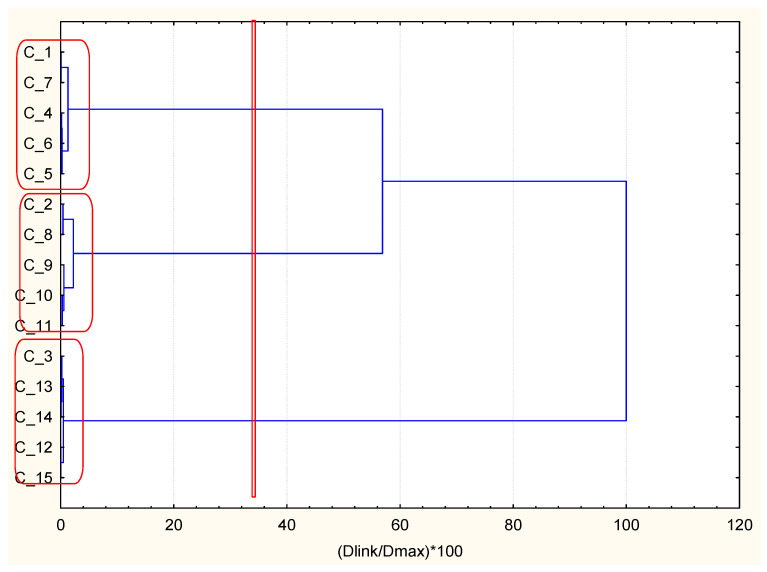
Hierarchical dendrogram for clustering of 15 occasionally selected patients with diabetes mellitus type 2 (DMT2) and arterial hypertonia (AH) as an underlying disease.

The selected group of 51 patients with arterial hypertonia underlying disease were selected for combinatorial k-means analysis. The clinical variables that were ranked in the first position with the minimum global variance criteria were BMI, HbA1c, and EAG (Table 2). The non-standardized values with the corresponding standard deviations are also shown in Table 2. It could be seen that cluster 1 has patients with low values of BMI, HbA1c, and EAG. Cluster 2 has high values of BMI and intermediate values of HbA1c and EAG, and cluster 3 has intermediate values of BMI and low values of HbA1c and EAG (Table 2 and Figure 2). The three clusters are better separated attending to the BMI factor. In order to quantify the descriptors that are able to better separate the clusters, a new parameter is defined taking into account the separation of the centroids relative to the dispersion of the data with:

(2) $$p_{i}^{\prime }=\max\left(\left|\left(x_{i}^{k1}-x_{i}^{k2}\right)\right|/\sigma_{i}^{12},\left|\left(x_{i}^{k1}-x_{i}^{k3}\right)\right|/\sigma_{i}^{13},\left|\left(x_{i}^{k2}-x_{i}^{k3}\right)\right|/\sigma_{i}^{23}\right)$$

where *x_i_* is the value of the property of descriptor *i* in the cluster *k*, and *σ_i_*^AB^ is calculated from the standard deviation in each cluster *σ_i_*.

(3) $$\sigma_{i}^{AB}=\sqrt{{\left(\sigma_{i}^{A}\right)}^{2}+{\left(\sigma_{i}^{B}\right)}^{2}}$$

It could be demonstrated that the parameter allows to compare the results with the test case study and allows to identify the descriptors that are more statistically different from the others. It is worth noting that the *p*’ parameter will provide the parameter *p* with the data of the test case.

In Table 2, the results for the BMI descriptor were depicted as applicable for clustering parting. The lowest value of *p*’ for the HbA1c and EAG descriptors specifies that these descriptors are less relevant for cluster separation.

Therefore, to illustrate the ability of the optimally selected new methodology, k-means partitioning descriptors for AH underlying disease hierarchical clustering was performed. Indeed, three well-defined clusters were obtained. They matched to the predefined groups of similarity (good health status (cluster 1), intermediate health status (cluster 2), and worsened health status (cluster 3) mainly with respect to the most optimal descriptor BMI; the other two descriptors reflect the glucose level, and they do not fit entirely into the separation of the health status mentioned above, but their discriminating probability (*p*’) is lower). It could be accepted that two major impacts determine the health status in this case–metabolic syndrome (dominant) and glucose level (less significant).

The analysis of CHD cases with combinatorial k-means clustering finding the minimum global variance is presented in Table 3 with BMI, creatinine, and cholesterol as the most appropriate descriptors.

**Figure 3:**
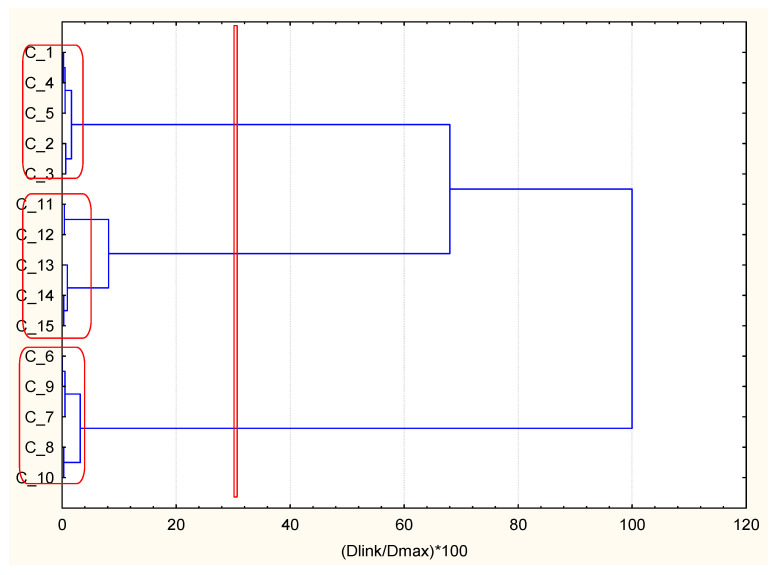
Hierarchical dendrogram for clustering of 15 occasionally selected patients with DMT2 and CHD as underlying diseases.

A similar control chart using hierarchical clustering with the descriptors for patients with DMT2 and ischemic heart disease (CHD) as an underlying problem is shown in Figure 3.

**Figure 4:**
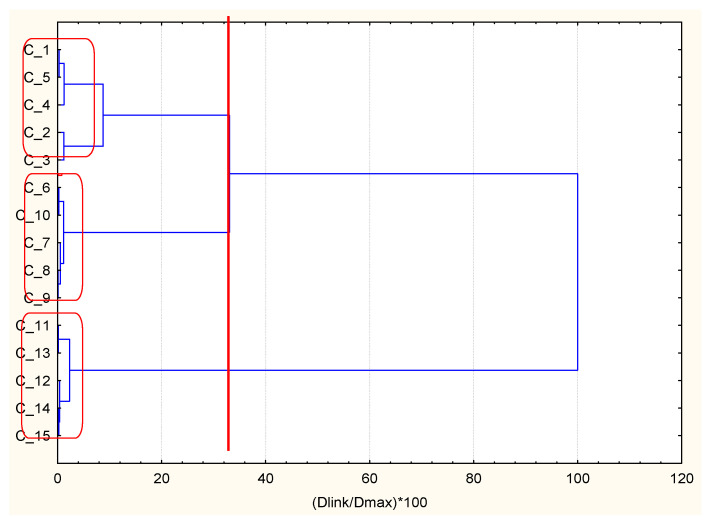
Hierarchical dendrogram for clustering of 15 occasionally selected patients with DMT2 and diabetic polyneuropathy (DPNP) as underlying diseases.

As readily seen, the separation of 15 randomly selected patients with DMT2 and CHD into three clusters is achieved and depicted in Figure 4 In this case, the optimal descriptor is the creatinine parameter (kidney function impact) since BMI and cholesterol (metabolic syndrome impact) are less discriminative parameters among all three chosen as optimal. Cluster 1 resembles good health status, cluster 2 resembles severe health status, and cluster 3 resembles intermediate health status. The combinatorial k-means analysis for patients with DPNP underlying disease contributes to the best parting of groups for BMI, HbA1c, and EAG parameters.

The separation into three clusters is well depicted by hierarchical clustering. The linkage resembles that of the case with AH underlying disease. BMI is the optimal descriptor (metabolic syndrome) since the glucose level parameters are of secondary importance. However, the health status for all three clusters is very similar (see the descriptor values in Table 4), and the subdivision into the three health status categories is quite conditional (especially cluster 1 and cluster 2). Cluster 3 includes the randomly selected patients with good health status. The combinatorial k-means analysis for DMT2 patients with DMA underlying disease gives the best separation of groups for k-means clustering of BMI, creatinine, and cholesterol descriptors (Table 5).

The last case (patients with DMT2 and DMA as underlying diseases) follows entirely the pattern of case 2 (patients with DMT2 and CHD as underlying diseases). It is worth noting that the three clusters with 15 randomly selected objects are obtained with creatinine as the optimal descriptor.

## 4. Conclusions

In this work, we investigated the ability of a new original k-means procedure to identify the descriptors that conduct clustering with the optimal separation between groups of object similarity. This procedure uses a combinatorial strategy with a selection of the descriptors that minimize the global variance obtained from the variance of all the descriptors in all the groups. The prediction separations are a proper method that could be applied for classifying descriptors and for better separation of the objects into groups. It was demonstrated that for this procedure, there is no precondition that all the variables need to be known for all the objects. We have shown that this technique has been applied to medical clinical data of patients with DMT2 with underlying diseases, where not all the information of all the variables is available for all the patients.

In summary, hierarchical clustering is confirmed to be efficient and suitable for screening studies and in combination with the new methodology to separate the objects of interest into a preliminary given number of clusters using the optimally determined descriptors. This is the first study that investigated the applicability of these models based on partitioning.

## Figures and Tables

**Table 1:** Probability to identify properly the *X*, *Y*, and *Z* descriptors from all 24,360 combinations as a function of the *p* parameter used for cluster separation.

*p*	Probability to Rank *X*, *Y,* and *Z* in First Position
2.2	3%
2.3	5%
2.4	10%
2.5	15%
2.6	21%
2.7	39%
2.8	45%
2.9	60%
3.0	75%
3.1	81%
3.2	87%
3.3	95%
3.4	96%
3.5	99%

All normal distributions have been performed with a σ = 1. The total number of descriptors is 26. Probability is calculated with 500 different repetitions of the combinatorial k-means clustering procedure.

**Table 2:** Variables of the best k-means clustering for arterial hypertonia underlying disease.

Descriptor	Cluster 1	Cluster 2	Cluster 3	*p*’
BMI	28 (1.2)	32 (1.6)	29 (1.3)	3.4
HbA1c	9.4 (0.8)	8.9 (0.7)	7.2 (0.6)	2.2
EAG	12.4 (1.3)	11.5 (1.1)	8.9 (0.9)	2.3

**Table 3:** Variables of the best k-means clustering for ischemic heart disease underlying disease.

Descriptor	Cluster 1	Cluster 2	Cluster 3	*p*’
BMI	30 (1.4)	31 (1.3)	29 (1.2)	1.8
Creatinine	62 (7)	135 (9)	83 (16)	6.2
Cholesterol	6 (0.6)	4.2 (0.4)	4.1 (0.9)	2.5

**Table 4:** Variables of the best k-means clustering for DPNP underlying disease.

Descriptor	Cluster 1	Cluster 2	Cluster 3	*p*’
BMI	32 (1.2)	34 (1.6)	30 (1.1)	3.4
HbA1c	9.4 (0.8)	9.0 (0.7)	7.3 (0.6)	2.1
EAG	12.4 (1.3)	11.7 (1.1)	8.9 (0.9)	2.2

**Table 5:** Variables of the best k-means clustering for diabetic microangiopathy (DMA) underlying disease.

Descriptor	Cluster 1	Cluster 2	Cluster 3	*p*’
BMI	30 (1.4)	31 (1.3)	29 (1.2)	2.0
Creatinine	62 (7)	135 (9)	83 (15)	6.2
Cholesterol	6 (0.6)	4.2 (0.4)	4.1 (0.9)	2.5

## Data Availability

Not applicable.
